# The evolution of ultraconserved elements with different phylogenetic origins

**DOI:** 10.1186/1471-2148-12-236

**Published:** 2012-12-05

**Authors:** Taewoo Ryu, Loqmane Seridi, Timothy Ravasi

**Affiliations:** 1 Integrative Systems Biology Lab., Division of Biological and Environmental Sciences & Engineering, Division of Applied Mathematics and Computer Sciences, King Abdullah University of Science and Technology, Thuwal, 23955-6900, Kingdom of Saudi Arabia; 2 Department of Medicine, Division of Medical Genetics, University of California, San Diego, 9500 Gilman Drive La Jolla, CA, 92093-0688, USA

**Keywords:** Ultraconserved elements, Developmental enhancers, Transcriptional regulatory networks, Genome evolution, Marine biology

## Abstract

**Background:**

Ultraconserved elements of DNA have been identified in vertebrate and invertebrate genomes. These elements have been found to have diverse functions, including enhancer activities in developmental processes. The evolutionary origins and functional roles of these elements in cellular systems, however, have not yet been determined.

**Results:**

Here, we identified a wide range of ultraconserved elements common to distant species, from primitive aquatic organisms to terrestrial species with complicated body systems, including some novel elements conserved in fruit fly and human. In addition to a well-known association with developmental genes, these DNA elements have a strong association with genes implicated in essential cell functions, such as epigenetic regulation, apoptosis, detoxification, innate immunity, and sensory reception. Interestingly, we observed that ultraconserved elements clustered by sequence similarity. Furthermore, species composition and flanking genes of clusters showed lineage-specific patterns. Ultraconserved elements are highly enriched with binding sites to developmental transcription factors regardless of how they cluster.

**Conclusion:**

We identified large numbers of ultraconserved elements across distant species. Specific classes of these conserved elements seem to have been generated before the divergence of taxa and fixed during the process of evolution. Our findings indicate that these ultraconserved elements are not the exclusive property of higher modern eukaryotes, but rather transmitted from their metazoan ancestors.

## Background

Large numbers of DNA elements (≥200 bp) exhibiting 100% similarity have been found to be conserved across several mammalian species [1,2]. Shorter ultraconserved elements (UCEs) longer than 50 bp and 100 bp have also been identified in several insect species and plants, respectively [3,4].

Since the discovery of UCEs, a lot of effort has been expended on elucidating their functions and to determine the reasons for their extreme conservation. UCEs are often located near genes implicated in transcription and developmental processes, splicing, and ion flow control across membranes [1,2,5-7]. In vivo analysis of the embryos of transgenic mice uncovered the transcriptional enhancer activities of UCEs targeting developmental genes and transcription factors (TFs) [8,9]. Depletion of UCEs among segmental duplications and copy number variations were also reported [10]. Single nucleotide polymorphisms (SNPs) in UCEs have been linked to cancer risk, impaired TF binding, and homeobox gene regulation in the central nervous system [11,12]. Nevertheless, homozygote embryo knockout experiments in mice revealed that deletion of ultraconserved elements can yield viable mice, suggesting the dispensability or functional redundancy of UCEs [13].

The origin and evolution of UCEs have also been also investigated. There is evidence that some UCEs originated from retroposons and stabilized in genomes after acquiring a function that benefitted the host [14]. Stephen et al. studied the evolution of UCEs in several vertebrate genomes and found that they were generated and expanded on a large scale during tetrapod evolution [15]. Other studies of the human genome showed that UCEs experienced strong purifying selection and were not mutational cold spots [16-18].

In this study, we investigated if evidence of the conservation of DNA elements could be found in primitive species, such as sponge and hydra, and if these conserved elements have similar functions as those previously reported for higher eukaryotes. We identified many UCEs across diverse phyla, including Porifera, Cnidaria, Arthropoda, Echinodermata, and Chordata, as well as a new type of short UCEs. By comparing distant species, we were able to identify new UCEs in human and fruit fly. Clustering the UCEs based on the sequence similarity unveiled lineage specificity and distinct functions outlined by protein domains of their flanking genes and DNA regulatory motifs. We concluded that each UCE group arose independently on a specific lineage and was “frozen” on the genome as a regulatory innovation after the divergence of specific taxa.

## Results and discussion

### Identification of ultraconserved elements across diverse taxa

We began our analysis by asking if there is evidence of ultraconservation in primitive species and, if so, how UCEs diverged during the process of evolution. We considered six species whose genomes were previously sequenced including demosponge (*Amphimedon queenslandica*) from the phylum Porifera, hydra (*Hydra magnipapillata*) and sea anemone (*Nematostella vectensis*) from the phylum Cnidaria, sea urchin (*Strongylocentrotus purpuratus*) from the phylum Echinodermata, fruit fly (*Drosophila melanogaster*) from the phylum Arthropoda, and human (*Homo sapiens*) from the phylum Chordata. We identified UCEs (≥50 bp) and shorter UCEs (≥30 bp) by pairwise comparison of the whole genomic sequences across six species.

**Figure 1:**
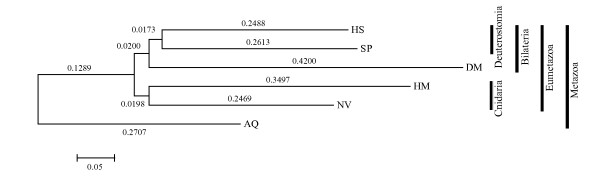
**Evolutionary relationships between analyzed species.** The JTT matrix-based method [61] is used to compute the evolutionary distances and the phylogenetic tree is constructed using the Neighbor-Joining method [62]. Bootstrapping values from 500 replicates are shown and selected taxon information is depicted on the right. Species abbreviations are as follows: AQ: *Amphimedon queenslandica* (sponge), DM: *Drosophila melanogaster* (fruit fly), HM: *Hydra magnipapillata* (hydra), HS: *Homo sapiens* (human), NV: *Nematostella vectensis* (sea anemone), SP: *Strongylocentrotus purpuratus* (sea urchin).

Unexpectedly, the number of identified UCEs and the size of some of them (11 UCEs ≥ 200 bp) were large considering the evolutionary distance between analyzed species. This result suggested the presence of UCEs in primitive species and across distant taxa (Table 1 and Figure 1). Most of the UCEs were found in hydra and sea anemone, which belong to the same phylum, Cnidaria. However, the exact reason for the predominance of UCEs in these species cannot be addressed until more genome sequences of species around this lineage become available and current genome assemblies are improved. Interestingly, the longest UCE (796 bp) was conserved in both sea anemone and human, two species that diverged approximately 892 million years ago [19]. We found that the number of UCEs and the evolutionary distance (Table 1 and Figure 1) between species are negatively correlated, an observation that is also the case for shorter UCEs.

**Table 1:** Identification of UCEs

	***A. queenslandica*(sponge)**	***N. vectensis*(sea anemone)**	***H. magnipapillata*(hydra)**	***D. melanogaster*(fruit fly)**	***S. purpuratus*(sea urchin)**	***H. sapiens*(human)**
*A. queenslandica*	-	2,135	669	43	108	9
*N. vectensis*	5,303	-	54,732	256	5,525	10
*H. magnipapillata*	1,300	97,669	-	125	400	0
*D. melanogaster*	75	5,440	478	-	188	27
*S. purpuratus*	537	43,707	5,498	1,129	-	19
*H. sapiens*	83	381	328	415	967	-

Columns and rows are sorted by the phylogeny as shown in Figure 1. Upper and lower triangles show the numbers of 100 % identical matches ≥ 50 bp and ≥ 30 bp between two species, respectively.

We noticed that a large number of conserved DNA elements that we identified overlapped in each species because the UCE-identification program, MUMmer, reported all maximal matches regardless of the overlap [20]. To minimize redundancy and facilitate downstream analysis, neighboring UCEs and short UCEs in each species were joined as non-overlapping ultraconserved regions (UCRs) (Additional file 1 and Additional file 2). The numbers of these non-overlapping UCRs (≥50 bp) were 30 for sponge, 64 for fruit fly, 673 for hydra, 56 for human, 3,807 for sea anemone, and 187 for sea urchin.

### Novel ultraconserved elements in human and fruit fly

As a benchmark for our UCE discovery pipeline, we examined how many UCEs that had been previously identified we were able to recover. Previously reported UCEs in human and fruit fly were aligned to their reference genome using Bowtie [21] to determine their exact locations in the current genome build (hg19 and dm3, respectively). The majority of known UCEs (all 481 elements from the human-mouse-rat alignment [1], 23,695 out of 23,699 elements from the *D. melanogasterDrosophila pseudoobscura* alignment, and all 126 elements from the *D. melanogasterAnopheles gambiae* alignment [3]) were successfully aligned. We then compared these elements with our UCR set. Unlike in the fruit fly where 42 out of 64 UCRs overlapped with data reported by Glazov et al. [3], we could not find any UCR in human that overlapped with previously reported UCEs [1] (Additional file 3).

**Figure 2:**
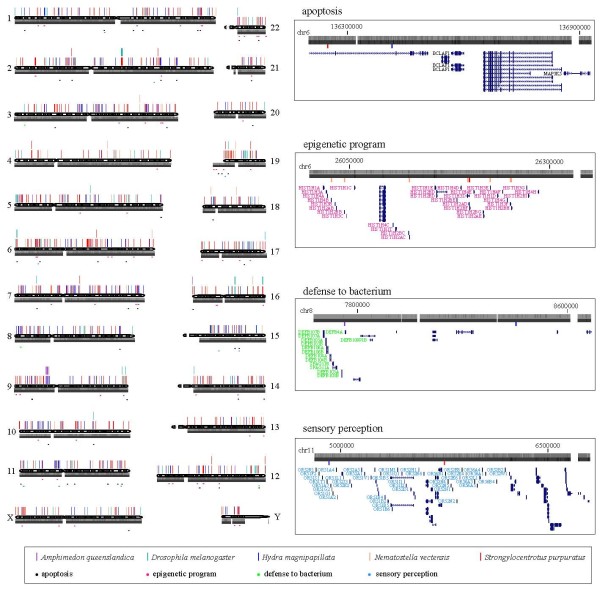
**UCEs in the human genome.** The short conserved elements (≥30 bp) are depicted above each chromosome and the UCEs (≥50 bp) are depicted above them. The conservation level of human DNA across primates or placental mammals is shown below each chromosome, where the darker color indicates more conservation. Species in which the human sequences are conserved and selected gene categories are labeled as indicated at the bottom. The selected region for each gene category is magnified for a detailed view in the right panel. R package quantsmooth [63] and the UCSC custom track [22] were used for the plot.

To understand this incongruence, we tested if our pipeline could recover known UCEs of the human-mouse-rat alignment with the same species list and length constraint (≥200 bp) of Bejerano et al. [1]. Our pipeline recovered 464 out of 481 known human UCEs that are conserved both in mouse and rat. The missing 17 known UCEs overlapped with repetitive regions, and these elements could not be recovered by our pipeline, which masks repetitive elements. Furthermore, the human UCEs that were conserved in mouse and rat identified by our pipeline did not also overlap with those newly identified in this study, suggesting that our pipeline works properly. The effect of the genome assembly version used for UCE identification was also negligible as explained above. On the other hand, our stringent repeat masking reduced the number of detectable known UCEs. The numbers of known UCEs were 304, 20,602, and 83 for human-mouse-rat, *D. melanogasterD. pseudoobscura*, and *D. melanogasterA. gambiae*, respectively, when we removed known UCEs with simple and known repetitive elements by repeat-masked chromosomes [22], CENSOR [23], and tandem repeat finder [24], the same criteria that we used in this study. However, the most important factor contributing to the identification of novel UCRs was the length constraint (50 bp for human) and species compared. To test this further, our human UCR set was divided into 50 bp sub-sequences, and then a search for these sub-sequences in the genomes of mouse and rat was conducted. Of 28 UCRs, one sub-sequence occurred in both the mouse and rat genomes with 100% similarity. On the other hand, the other 28 UCRs were not conserved in both species, suggesting that those sequences were no longer under strong selective pressure in rodents and could therefore not be identified by the traditional human-mouse-rat alignment (Additional file 3). Indeed, large portions of identified human UCEs are positioned in less conserved loci in placental mammals (Figure 2), which further supports our findings of novel highly conserved DNA elements in model organisms.

### UCR clusters arose independently

We then sought evidence for if UCRs from the same or different species share similarity. Considering the short length of UCRs and also assuming that distal regions of ultraconserved elements have higher mutation rates than proximal regions [15,25,26], we analyzed UCRs and their 50 bp-flanking sequences. In all, 4,817 UCRs with flanking sequences from all species were clustered, and orthologous and paralogous UCRs were defined. This yielded 61 clusters, of which the largest cluster consisted of 1,168 UCRs from hydra, sea anemone, and sea urchin (Additional file 4).

Although there are large numbers of UCRs across different taxa, we found that UCRs share sequence similarities and that each cluster of UCRs has a distinct species composition. Moreover, Cnidarian UCRs show a tight association, while human UCRs are largely clustered together with those of sea urchin and/or fruit fly (Additional file 4). Gain of essential functions for the survival of the species in ancestral sequences might contribute to the conservation of the sequence in a specific lineage [14]. Another possible explanation would be that even if the ancestral sequences were not beneficial to the species, random sampling contributed to the elimination of other alleles and the fixation of these sequences in the downsized population, creating a new lineage, due to natural catastrophe or population migration, referred to as a “genetic drift” or “population bottleneck” [27]. Although further study is required to explain the immutability of UCEs after lineage divergence and sequence fixation across a long evolutionary history, we cannot rule out this possibility. It also should be noted that the absence of UCRs in species from the same lineage does not necessarily mean that those UCRs disappeared in those species but rather that they may exist as derivative sequences by mutation [2,15,28,29].

**Figure 3:**
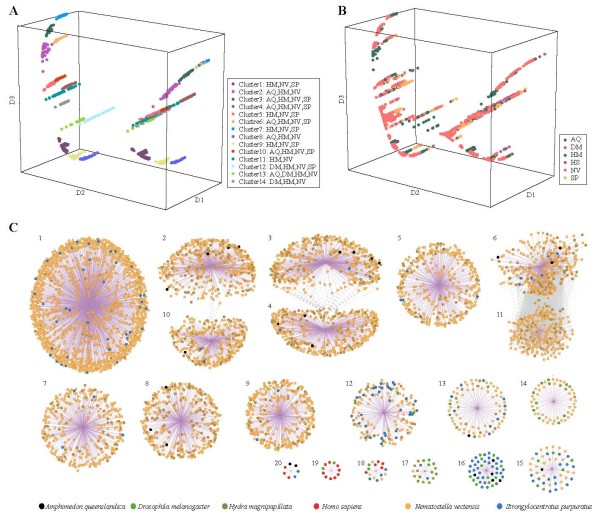
**UCR clusters arose independently during evolution.A** and **B**. The 5-mer composition of UCRs and 50 bp-flanking sequences were taken as input for the Minimum Curvilinear Embedding analysis and the top three dimensions are depicted here. Clusters and species are marked with different colors as indicated in the inset on the lower right corner. **C**. UCR cluster relationships. Each node represents a UCR with flanking sequence. The similarity between nodes in a same cluster is omitted to avoid extreme density. A cluster centroid is made instead and connected to the components to show membership within the cluster (purple lines). The gray lines show sequence similarities between nodes in different clusters. Clusters with fewer than 7 nodes are not shown. UCRs from sea anemone predominate in this figure due to the large number (3,807 among all 4,817 UCRs).

As shown in Figure 3A and Additional file 5, UCR clusters are clearly separated in a Minimum Curvilinear Embedding (MCE) plot [30], although species is not a good factor to distinguish UCRs (Figure 3B). Short UCRs (≥30 bp) also followed a similar pattern. Interestingly, some clusters have nearly symmetric elements on the MCE plot and it turns out that they are partially reversed complementary sequences.

Network topology demonstrates the relationship between these UCR clusters, where some clusters are connected due to the sequence similarity between components, although most clusters do not share sequence similarity with others and have unique species composition (Figure 3C). Thus, the UCRs of each cluster may have their own independent origin in a specific lineage.

### The neighboring genes of UCRs have distinct functions

**Figure 4:**
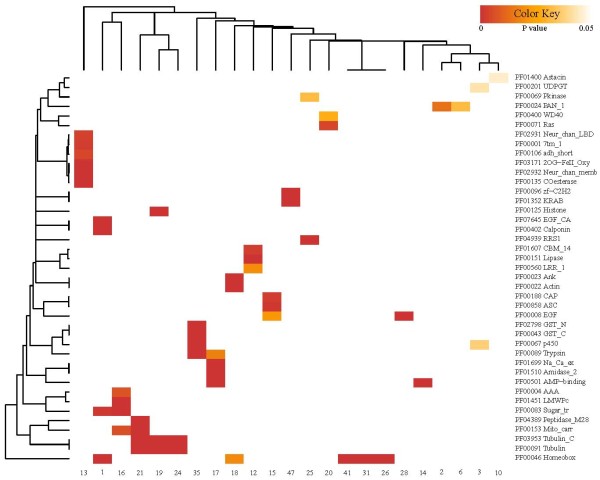
**Protein domain enrichment of UCR flanking genes.** Association of domains (rows) or clusters (columns) is depicted in dendrograms on the right and upper side of the heatmap, respectively. Only clusters having at least 10 genes were analyzed. Domains whose p-value. <0.05 in at least one cluster and that occurred in at least three nearby genes are shown on the heatmap for visualization purpose.

UCEs are often flanked by developmental genes, TFs, ion channels, or splicing factors [5,7]. We investigated the functions of each cluster’s nearby genes. Due to the paucity of functional annotations of genes and the short length of genome scaffolds in non-model species (Additional file 6), we focused our analysis on the protein domains of nearby genes within 100 kb from UCRs. Neighboring genes to UCR clusters span a spectrum of statistically significant protein domains. However, each cluster is enriched with a distinct set of domains (Figure 4).

Ion channel and transporter domains are the predominant categories; they appear in many clusters composed of various species. Neurotransmitter-gated ion channels and sodium or calcium ion exchanger genes are overrepresented in clusters 13, 15, and 17, whose UCRs are conserved in all species considered here but human (Figure 4 and Additional file 4). Cation transporters are identified in cluster 30, which consists of human and fruit fly UCRs. Sugar transporters and mitochondrial carrier domains that transport various molecules across membranes are enriched in clusters 1, 16, and 21. These observations are probably because ion channels and transporters are crucial in all living organisms for the maintenance of water, salt, and nutrient homeostasis as well as for electric signal transmission in neuronal and muscle cells [31].

The homeobox domain, part of the TFs that act during the developmental process, is enriched in five clusters. This domain is found in all six species, with three of the five enriched UCR clusters composed of UCRs from human and fruit fly, one from fruit fly and sea urchin, and the last cluster from hydra, sea anemone, and sea urchin. Fruit fly genes regulating developmental programs ranging from axis patterning to molting, such as *bicoid*, *fushi tarazu*, and ecdysone receptor, are also found in several clusters, even those without significant domains.

Histones are overrepresented in cluster 19, which consists of sea anemone and sea urchin UCRs. Evidence that chromatin-related genes flank conserved elements in human (Additional file 7) and from other studies [32,33] suggest that there is a liaison between conserved elements and epigenetic control mechanisms.

Detoxification domains such as cytochrome p450, UDPGT, and GST are enriched in cluster 3 and cluster 35. Cluster 3 consists of UCRs from sponge, hydra, sea anemone, and sea urchin; cluster 35 consists of UCRs from fruit fly and human. These enzymes are important to catalyzing and eliminating endogenous and exogenous substrates and therefore to providing a healthy environment for the cellular system [34]. This remarkable linkage between UCRs and detoxification mechanisms has not previously been reported to our knowledge.

Further analysis of UCRs (≥50 bp) and short UCRs (≥30 bp) in human reveals similar but more interesting properties in terms of nearby gene functions and species conservation (Additional file 7 and Additional file 8). Genes acting in various developmental processes are highly enriched near the UCRs in human that are also conserved in fruit fly and sea urchin. To our surprise and contrary to previous studies, few genes related to development are enriched near the human sequences conserved in sponge, hydra, or sea anemone. Expansion of the relationship between developmental programs and UCRs in human, fruit fly and sea urchin (Figure 1 and Additional file 7 and Additional file 8) implies that the association of conserved sequences with the regulation of developmental genes started or expanded after the divergence of the Bilateria lineage from the metazoan stem. Our UCR clustering results bolster this hypothesis (Figure 4). Four out of five UCR clusters that have overrepresented homeobox domains of nearby genes come from human, fruit fly, and sea urchin.

Interestingly, genes surrounding short UCRs are enriched with epigenetic program-related genes (Figure 2 and Additional file 7). Short UCRs conserved in human and in fruit fly, hydra, sea anemone, or sea urchin are located near histone gene clusters across several chromosomes. Furthermore, many important epigenetic regulators are also found near elements conserved in sponge, hydra, sea anemone, or sea urchin. These include histone demethylases (KDM3B, KDM4C, KDM5C, and KDM5D), histone acetyltransferases (EP300 and KAT7), histone deacetylases (HDAC2 and HDAC10), retinoblastoma-like protein (RBL1), polycomb ring finger oncogene (BMI1), chromodomain helicase (CHD8), and components of the chromatin remodeling complex, SWI/SNF (SMARCA2, SMARCB1, SMARCC2, and SMARCD3). Taken together with the previously suggested relationship between highly/ultraconserved elements and epigenetic control [15,32,33], our results suggest an interesting hypothesis that epigenetic control mechanisms have tight relationships with conserved DNA sequences and that they might have coevolved from metazoan ancestors rather than recently developed.

Genes implicated in apoptosis, olfactory reception, and defense mechanisms are also enriched near DNA elements conserved in sponge, hydra, or sea urchin (Figure 2 and Additional file 7 and Additional file 8). Our analysis suggests that genomes preserve ancestral sequences well, and these ancestral sequences might have coevolved with a diverse set of essential genes. When and how genes and conserved elements initiated their relationships remains unclear and the mechanism for such an association needs to be further elucidated. However, our analysis expands the repertoire of conserved genomic elements that are possible regulatory elements.

### UCRs are enriched with binding sites for developmental TFs

**Figure 5:**
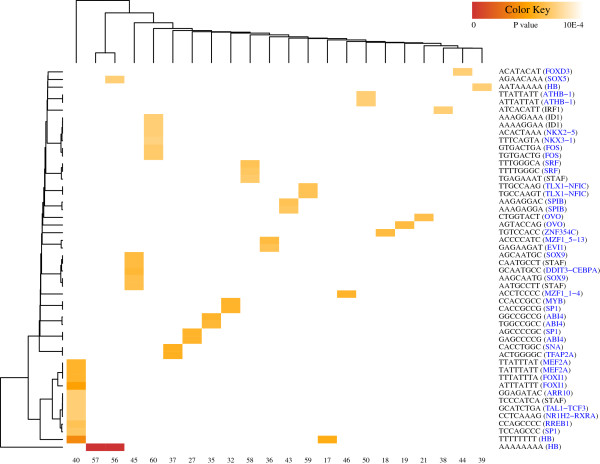
**Oligomer enrichment on UCRs.** Each cluster (column) shows distinct TFBS patterns. Association of 8-mers and clusters is depicted in dendrograms. Only 8-mers with p-value<1E-4 are shown by the heatmap. The most related TFBSs of each 8-mer are shown in the brackets on the right side. TFs with developmental functions indicated by NCBI Gene [64] or GeneCards [65] are colored blue.

The enhancer activities of UCEs have been reported by several studies [8,9]. To investigate the possibility that these enhancer activities were also conserved in primitive species, we identified significantly overrepresented oligomers and related TF binding sites (TFBSs) for each UCR cluster (Figure 5).

Among 31 TFs that had significant 8-mer matches, 28 were implicated in developmental processes and many were homeobox TFs. Binding sites of homeobox TFs on UCEs near the developmental genes in higher eukaryotes have been identified [35-37], although our clustering results identified various nearby gene categories that were not limited to developmental genes. Prevalent occurrence of developmental TFBSs regardless of cluster and species may be an indication that extensive binding of developmental TFs on UCEs existed in metazoan ancestors and these TFs regulated various nearby genes to coordinate developmental functions. These may have contributed to the strong selective pressure on UCEs that function as regulatory sequences.

## Conclusions

Genomes are dynamic entities and are under selective evolutionary pressure from mutation and fixation. Beneficial or neutral mutations in the ancestors of specific lineages are maintained in the population and vertically transferred to descendants [38]. However, these dynamic and selective pressures are not applied uniformly across the whole genome [16,39,40]. Deleterious mutations in essential regions are corrected in a population [15,16]. Sequence conservation thus implies that the function of the sequence is essential. Despite controversy about the indispensability of ultraconserved elements [13,41], much work has demonstrated various vital functions of such elements [5,6,8-10].

As more genomes from various taxa are being sequenced, the opportunity to understand genome conservation and usage increases. Here, we compared genome sequences ranging from primitive aquatic to higher terrestrial species and described for the first time a number of novel UCEs present in primitive species as well as previously uncharacterized UCEs in human and fruit fly. We observed that UCEs cluster by sequence similarity and each cluster has distinct patterns of species composition. These UCEs also exhibited specific biases toward the function of nearby genes and oligomer compositions of the UCE sequences, suggesting that each group of UCEs was generated in the common ancestors of specific lineages and fixed during the evolution of descendants. Although a more detailed functional analysis of UCEs cannot currently be conducted due to the nature of the short draft sequences and because gene functions of non-model species have been less studied, our analysis suggests that UCEs harbor important sequence features, such as binding sites of developmental TFs to coordinate the expression of essential genes, which is why they were readily conserved over the long course of evolution.

## Methods

### Data preparation

Genome sequences, gene annotation, and protein sequences were downloaded from the UCSC database for human (assembly version: hg19) and fruit fly (assembly version: dm3), and each genome project for sponge (assembly version as of 5 Aug 2010) [42], hydra (assembly version as of 28 Jan 2009) [43], sea anemone (assembly version as of 26 Oct 2005) [44], and sea urchin (assembly version as of 13 Oct 2006) [45].

### Phylogenetic analysis

First, we identified single copy genes from each of six species under investigation to infer their phylogenetic relationships. This approach had been used previously in other studies to avoid the paralogy issue [44,46,47]. Inparanoid was used to identify orthologs and paralogs between species pairs [48]. Only the longest peptide was used when multiple transcripts came from the same gene. We identified 472 single-copy genes that were found to be largely involved in ribosome, spliceosome, or proteasome pathways. Gene sequences were aligned using MUSCLE [49] and the evolutionary distance and phylogenetic tree were obtained using MEGA5 [50]. The phylogenetic tree reveals the overall relationship between six species, which was in agreement with the known classification of these lineages (Figure 1) [45,51,52].

### Identification of ultraconserved elements

To identify UCEs for all species pairs, we masked repetitive sequences in the scaffolds of sponge, hydra, sea anemone, and sea urchin using CENSOR [23] and tandem repeats finder [24]. Repeat-masked chromosomes from the UCSC database were used for human and fruit fly [22]. To identify non-gapped conserved elements between two species, we used MUMmer, which rapidly aligned long sequences and detected exact matches using the suffix tree algorithm, with the *maxmatch* option to compute all maximal identical matches regardless of uniqueness [20]. Both forward and reverse complement matches were reported. Identical matches equal to or longer than 50 bp were identified, and ≥30 bp matches were also identified for incidental analysis. Identified UCEs were further masked using CENSOR and tandem repeat finder again. It should be mentioned that this stringent repeat-masking process may have deleted potential UCEs containing repetitive elements.

Two UCEs were joined if they overlapped, and this merging process was repeated until no two UCEs overlapped (Additional file 1 and Additional file 2). Fifty base flanking sequences on both sides of merged UCEs were retrieved using the custom python script.

### Clustering of ultraconserved elements

Merged ultraconserved elements with flanking sequences were grouped by sequence similarity. Pairwise alignment of all sequences was computed using BLASTN [53]. The score density, i.e. the BLAST bit-score divided by the alignment length, was used as the similarity measure. Sequences were clustered using the Markov cluster (MCL) algorithm [54] with default parameters (Additional file 4). In the Minimum Curvilinear Embedding (MCE) analysis [30], 5-mer compositions of the sequences were used as features. In particular, we used the new singular-value-decomposition-based algorithm to implement MCE [55], using the Matlab code provided on the author’s website (https://sites.google.com/site/carlovittoriocannistraci/home). The embedding was performed without centering the minimum curvilinear kernel (non-centered MCE).

### Nearby genes analysis

Flanking genes within 100 kb of the merged UCEs were obtained from all species under study. For human and fruit fly, we used the gene models from RefSeq [56]. We used the gene models from the respective genome sequencing projects of the non-model metazoans.

Pfam domains of nearby genes were annotated using Interproscan [57] for functional analysis of UCEs. For each domain in each UCR cluster, the domain enrichment of nearby genes within 100 kb of UCRs was calculated using cumulative hypergeometric distribution:

$$\text{P=}\;\sum_{i=d}^{\text{min}\left(D,g\right)}\frac{\left(\frac{D}{i}\right)\left(\frac{G-D}{g-i}\right)}{\left(\frac{G}{g}\right)},$$

where *G* is the total number of genes from the species pool in the cluster, *g* is the number of selected nearby genes in the species pool in the cluster, *D* is the number of occurrences of the domain in the species pool in the cluster, and *d* is the number of occurrences of the domain in the selected nearby genes in the species pool of the cluster.

Gene ontology enrichment of the nearby genes was analyzed using DAVID [58]. Considering that human has the most comprehensive biological process terms and nearly nothing is annotated in non-model species, only human UCRs and their nearby genes were analyzed.

### Motif analysis

A representative sequence of each cluster was generated using MUSCLE [49] and the seqinR package in R [59]. To assess the statistical significance of overrepresented 8-mers, we generated a 10 kb background sequence for each cluster. The background sequence was a combination of segments chosen randomly from all genomes, and each genome contributed to the background with an amount equal to the ratio of its species in the cluster composition. A cumulative binomial probability of observing the given number of the oligomer or more in each cluster was then computed as follows:

$$F\left(x|n,p\right)=1-\sum_{i=0}^{x-1}\left({}_{i}^{n}\right)p^{i}{\left(1-p\right)}^{n-i,}$$

where *x* is the number of occurrences of the oligomer, *n* is the sample size, i.e. sequence length - oligomer size + 1, and *p* is the probability of observing such an oligomer in the random background sequence. Related TFs for oligomers were identified using STAMP [60].

## Abbreviations

UCE: ultraconserved element; TF: transcription factor; SNP: single nucleotide polymorphism; AQ: *Amphimedon queenslandica*; DM: *Drosophila melanogaster*; HM: *Hydra magnipapillata*; HS: *Homo sapiens*; NV: *Nematostella vectensis*; SP: *Strongylocentrotus purpuratus*; UCR: ultraconserved region; MCE: Minimum Curvilinear Embedding; TFBS: TF binding site.

## Competing interests

The authors declare that they have no competing interests.

## Authors’ contributions

TR1 (Taewoo Ryu) and TR2 (Timothy Ravasi) conceived the overall study; TR1 and LS performed the data analysis; TR1, LS, and TR2 drafted the manuscript; all authors read and approved the final manuscript.

## Supplementary Material

Additional file 1 UCR information (≥50 bp) for each species. Click here for file

Additional file 2 Short UCR information (≥30 bp) for each species. Click here for file

Additional file 3 Comparison between identified UCRs and previously known fruit fly UCEs. Click here for file

Additional file 4 Clustering results of UCRs with 50 bp flanking sequences. Click here for file

Additional file 5 Visualization of the UCE clusters by minimum cuvilinear embedding. Click here for file

Additional file 6 Distribution of scaffold length for non-model species. Click here for file

Additional file 7 **Gene ontology enrichment of nearby genes within 50 kb, 100 kb, and 200 kb from short UCRs.** Only biological process terms are used. Click here for file

Additional file 8 **Gene ontology enrichment of nearby genes within 50 kb, 100 kb, and 200 kb from UCRs.** Only biological process terms are used. Click here for file
