# Supplementary figures and images for: The evolution of ultraconserved elements with different phylogenetic origins
Source: BMC Evol Biol. 2012 Dec 5;12:236. doi: 10.1186/1471-2148-12-236 (PMC3556307; doi:10.1186/1471-2148-12-236)

scaffold length

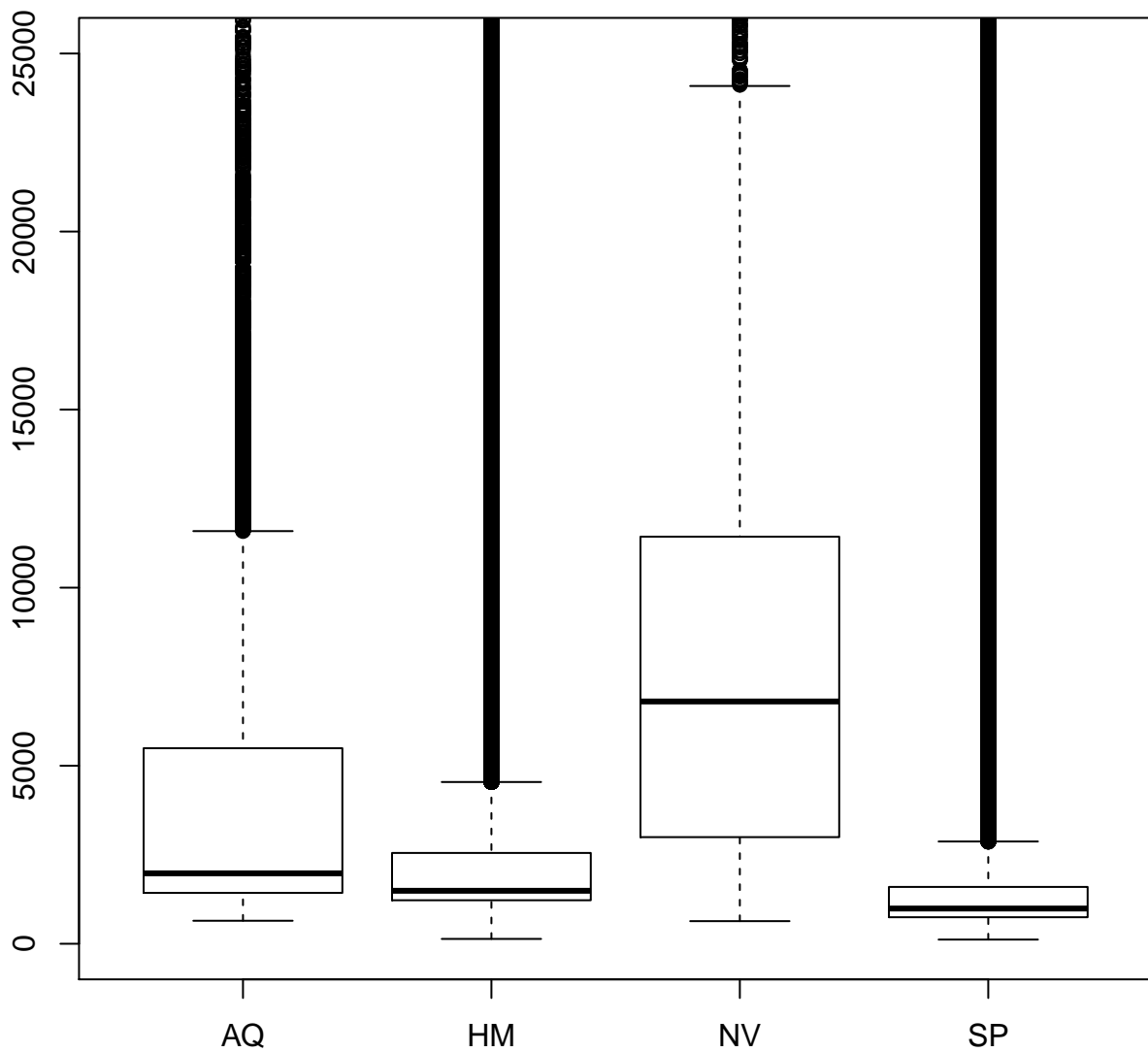

Supplement: Additional file 6 — Distribution of scaffold length for non-model species. [file 1471-2148-12-236-S6.pdf]
